# Craving for a Robust Methodology: A Systematic Review of Machine Learning Algorithms on Substance-Use Disorders Treatment Outcomes

**DOI:** 10.1007/s11469-024-01403-z

**Published:** 2024-10-04

**Authors:** Bernardo Paim de Mattos, Christian Mattjie, Rafaela Ravazio, Rodrigo C. Barros, Rodrigo Grassi-Oliveira

**Affiliations:** 1 Developmental Cognitive Neuroscience Lab, Pontifical Catholic University of Rio Grande do Sul (PUCRS), Porto Alegre, Rio Grande do Sul Brazil; 2 Machine Learning Theory and Applications Lab, School of Technology, Pontifical Catholic University of Rio Grande do Sul (PUCRS), Porto Alegre, Rio Grande do Sul Brazil; 3 Aarhus University - Translational Neuropsychiatry Unit, Department of Clinical Medicine, AU, Entrance A, Palle Juul-Jensens Blvd. 11, 6th floor, Aarhus, 8200 Denmark

**Keywords:** Machine learning, Substance-related disorders, Treatment outcome, Prediction models, Systematic review

## Abstract

Substance use disorders (SUDs) pose significant mental health challenges due to their chronic nature, health implications, impact on quality of life, and variability of treatment response. This systematic review critically examines the application of machine learning (ML) algorithms in predicting and analyzing treatment outcomes in SUDs. Conducting a thorough search across PubMed, Embase, Scopus, and Web of Science, we identified 28 studies that met our inclusion criteria from an initial pool of 362 articles. The MI-CLAIM and CHARMS instruments were utilized for methodological quality and bias assessment. Reviewed studies encompass an array of SUDs, mainly opioids, cocaine, and alcohol use, predicting outcomes such as treatment adherence, relapse, and severity assessment. Our analysis reveals a significant potential of ML models in enhancing predictive accuracy and clinical decision-making in SUD treatment. However, we also identify critical gaps in methodological consistency, transparency, and external validation among the studies reviewed. Our review underscores the necessity for standardized protocols and best practices in applying ML within SUD while providing recommendations and guidelines for future research.

**Supplementary Information:**

The online version contains supplementary material available at 10.1007/s11469-024-01403-z.

Substance use disorders (SUDs) are characterized by their chronic course (National Institute on Drug Abuse (NIDA), 2023) and a spectrum of adverse consequences, presenting multifaceted challenges in mental health. SUDs are one of the mental health disorders that most often require both outpatient and inpatient treatment (Levola et al., 2021; Andersson et al., 2019; Brackett et al., 2022), with impact in mental, social, and physical health markers. These include increased mortality rate (Decker et al., 2017; Hjemsæter et al., 2019), high rates of relapse (McLellan et al., 2000; National Institute on Drug Abuse (NIDA), 2023), rehospitalization (Di Giovanni et al., 2020), and overall diminished global quality of life (Castaldelli-Maia et al., 2023).

The limited effectiveness of current interventions and treatments further compounds the issues faced, as low adherence rates and limited availability of public health services in certain regions lead to a substantial number of individuals with SUD not receiving adequate care (Ritter et al., 2019) according to data collected from the US, Italy, Germany, Hungary, Latvia, Poland, Spain, Australia, and the Netherlands.

This complex nature of SUDs, coupled with the sheer volume of individuals affected, presents a significant challenge in both research and clinical practice. The vast amount of data generated in these settings encompasses not only patient clinical histories and specific treatment outcomes, but also spans biological, genetic, epigenetic, and advanced imaging data, making it a rich yet intricate environment of data. Deciphering clinically relevant patterns and correlations among these diverse data sources is challenging yet crucial for gaining a deeper understanding of SUDs and devising more effective management strategies.

Machine learning (ML), a pivotal branch of artificial intelligence, is revolutionizing the approach to complex data-driven challenges across various disciplines, including psychiatry  (Jayatilake & Ganegoda, 2021). In psychiatry, ML is applied using several paradigms: supervised learning, where data is associated with a known outcome, such as data from patients with or without SUD; unsupervised learning, which discovers patterns in data without predefined labels; semi-supervised learning, which uses both labeled and unlabeled data; and reinforcement learning, which learns actions based on rewards and penalties. ML models are used in different tasks such as classification, diagnostic categorization, and regression for predicting length of stay. Commonly used algorithms include decision trees, neural networks, and ensemble methods like random forests.

In SUD, ML algorithms are increasingly being recognized for their potential to revolutionize both the prediction and management of clinical outcomes (Yang et al., 2023; Heberle et al., 2024). As the body of research grows (Chhetri et al., 2023; Garbin et al., 2023; Mak et al., 2019), it becomes clear that these algorithms are beneficial at uncovering intricate patterns within extensive datasets.

This ability is crucial in psychiatry, giving us predictive power over risk behaviors — such as relapse  (Roberts et al., 2022), susceptibility to addiction — such as epidemiological research  (Jing et al., 2020), as well as in determining effective treatment paths — such as treatment prescription  (Baucum et al., 2023). ML not only aids in identifying these patterns but also supports clinicians in making more informed decisions, tailoring treatment plans to the individualized needs of patients. However, the adoption of ML in the study of SUDs is not without its challenges. Methodological rigor is paramount to ensure the reliability and validity of findings. Common pitfalls in ML studies, such as data leakage, overfitting, and underpowered analyses, can significantly distort outcomes, leading to misleading interpretations and conclusions.

Despite the potential of ML, the current literature is fragmented and often quickly outdated given ML’s rapid development and emergent status, failing to adequately incorporate recent advancements in both ML technology and SUD treatment modalities while providing a critical examination of the methodological robustness behind the studies. This systematic review aims to address this by providing a comprehensive synthesis of current knowledge, highlighting the strengths and limitations of ML in the field of SUDs.

## Methods

This systematic review was conducted in accordance with the recommendations outlined in the Cochrane Handbook for Systematic Reviews of Interventions (Higgins et al., 2023), which guided the development of the search strategy, selection of studies, and data extraction. To ensure transparent and comprehensive reporting of the review process and findings, we adhered to the Preferred Reporting Items for Systematic Reviews and Meta-Analyses (PRISMA) guidelines (Moher et al., 2009; Page et al., 2021) and its extension for reporting literature searches in systematic reviews (PRISMA-S) (Rethlefsen et al., 2021). The review protocol was registered on the International Prospective Register of Systematic Reviews (PROSPERO) with the following identifier: CRD42023431546 (Available from: https://www.crd.york.ac.uk/prospero/display_record.php?ID=CRD42023431546).

### Identification and Selection of Studies

The literature search and screening of titles, abstracts, and full-text articles were independently conducted by three investigators, following the eligibility criteria specified in the “Eligibility” section. The electronic search was carried out on June 22, 2023, across several databases and updated on the 8th of August 2024. Records were retrieved with no date restrictions. We selected PubMed, Embase, Web of Science, and Scopus for our systematic review as they are among the most comprehensive and widely used databases in medical and scientific research. The search strategy was tailored to each database, and all search keys are available in the Supplementary Materials (Table S1).

### Eligibility

Eligible studies were cross-sectional, longitudinal, and case-control designs. We excluded other types of publications, such as reviews, background studies, and preclinical research, focusing solely on studies involving human participants diagnosed with SUDs, irrespective of age, gender, or ethnicity. The search criteria targeted research studies that have implemented machine learning or deep learning models to measure and analyze treatment outcomes in samples of substance-use-disorder individuals. Any study reporting on outcomes such as treatment adherence, relapse, readmission, or SUD severity was considered for inclusion. A detailed list of all studies excluded from the final review, along with the specific reasons for their exclusion, has been made available in the Supplementary Materials (Table S2).

### Data Extraction

Duplicates were excluded before the selection of the studies. Titles and abstracts were screened independently by three main reviewers (“CM,” “RR,” and “BPM”) to assess the initial eligibility. The full texts were then read to decide which were included in the systematic review. In cases of discrepancies during screening or full-text review, two senior reviewers (“RGO” and “RCB”) were consulted. These senior reviewers resolved conflicts by reviewing the disputed articles together and made a decision based on our predefined inclusion criteria. After screening the initial articles, the full texts were read to decide which were included in the systematic review. The following data were extracted from all included studies by the three main reviewers: “title,” “country in which the study was conducted,” “aim of study,” “study design,” “intervention description,” “outcomes” (for both ML algorithms and SUD treatment), “population description,” “substance,” “diagnostic criteria,” “comorbidities,” “total number of participants,” “inclusion criteria,” “exclusion criteria,” “model strategy,” “models used,” “input data,” “evaluation method,” “model performance,” “most relevant features,” and “limitations.”

### Methodological Quality and Risk of Bias Assessment

The assessment of methodological quality and risk of bias was conducted independently by the three main reviewers at both the study and outcome levels, using two distinct instruments recommended by the EQUATOR network (EQUATOR Network, 2024): the MI-CLAIM checklist (Norgeot et al., 2020) and the CHARMS checklist (Moons et al., 2014).

The MI-CLAIM checklist, which is focused on clinical artificial intelligence modeling, was employed to evaluate the methodological rigor and transparency specific to machine learning models. In contrast, the CHARMS checklist is designed for prediction modeling studies and comprehensively evaluates model development, validation, and impact assessment. These tools provided nuanced insights into each study’s methodological strengths and limitations. The understandings garnered from these evaluations were integral to our interpretation of the overall findings and the discussion of potential biases, enriching our understanding of the studies’ contributions to the field.

**Fig. 1:**
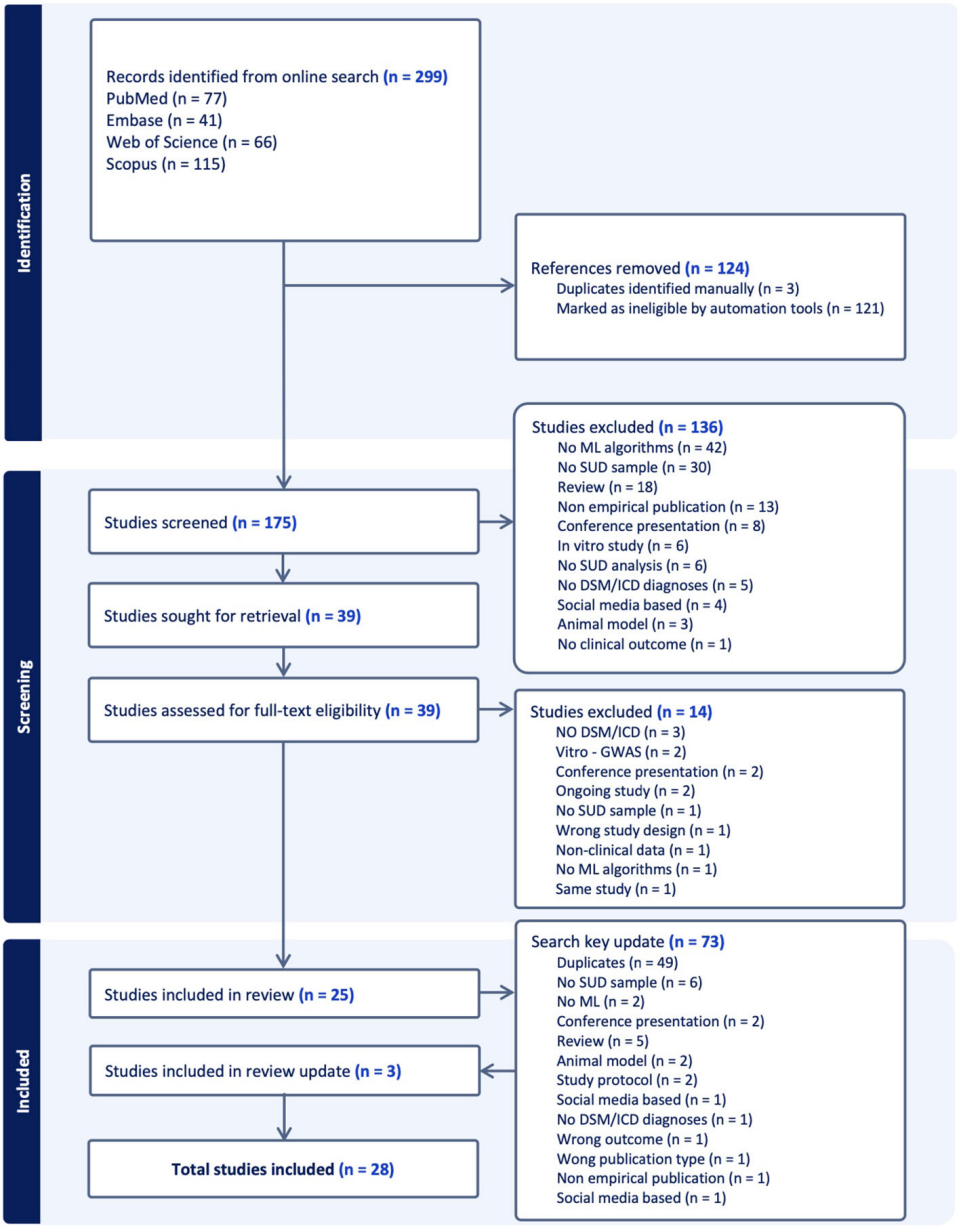
Preferred Reporting Items for Systematic Reviews and Meta-Analyses (PRISMA) flow diagram of the included registries

## Results

### Search Results

The initial search yielded 299 records. After removing duplicates ($n=124$), we screened 175 studies by reviewing the title and abstract. One hundred thirty-six records were excluded, and the remaining studies ($n=39$) were full-text reviewed, for which we applied the exclusion criteria. Fourteen were excluded following the determined exclusion criteria, leaving a total of 25 studies that were included in the systematic review. The updated search yielded 73 records. After removing duplicates ($n=49$), we screened 24 studies by reviewing them in full-text. A total of 28 studies were included in the systematic review. Detailed information about the inclusion process and excluded studies are presented in Fig. 1. A detailed list of all studies excluded from the final review, along with the specific reasons for their exclusion, has been provided in the Supplementary Materials (see Table S2). Such a table aims to ensure transparency and reproducibility of the study selection process.

### Characteristics of the Reviewed Studies

Table 1 shows the main information of each study, including the analyzed substance, the investigated outcome (output of the ML model), the study design, sample, origin, population description (general information; age; SUD diagnostic criteria), features used as input for the model prediction, and the ML models used.

The range of publication was between 2014 and 2024 (Steele et al., 2014; Heberle et al., 2024). Eighty-two percent of the studies analyzed data from USA samples. Data from Brazil, Canada, China, Italy, and Australia were explored by one study each. Seventy-nine percent of the studies had a cross-sectional study design, while 21% had a longitudinal design. Forty-six percent used data from trials of interventions for SUD patients, such as the COMBINE trial (Roberts et al., 2022).

**Table 1:** Characteristics of the included studies

Ref.	Subs.	Outcome	Design	Sample	Origin	Population description	Input features	ML models
Eddie et al. (2024)	Alcohol; opioid	Adherence	L	15873 ind.	USA#	Individuals on treatment, ages $12-17$ ($34.5\%$), $18-29$ ($40.2\%$), $30+$ ($25.3\%$);$48.7\%$ female, $51.3\%$ male; $10.6\%$ Alaska Native, $5.8\%$ Asian, $27.4\%$ Black, $22.1\%$ Hispanic,$1.9\%$ Native American, $0.7\%$ Pacific Islander, $29.5\%$ White, $2.0\%$ Other; $18.4\%$ unemployed; $22.9\%$ in school; $15.7\%$ homeless before treatment; DSM-IV for SUD	Demographic; psychosocial; treatment-specific	RF; GLLM
Heberle et al. (2024)	Cocaine	Craving severity	L	402 ind.	Brazil	Women on detoxification treatment with completed baseline and follow-up assessments at 3 weeks; $\ge 18$ years, $31.91\pm 8.94$ non-severe withdrawal; $33.57\pm 9.28$ severe withdrawal; SCID for CUD	CSSA score of the first week of treatment; ASI-6; CTQ	KNN; SVM; LR; Light GBM; NB; RF
Houghton et al.(2023)	Cocaine, cannabis, opioid	Identify SUD	CS	160 ind.	USA	Individuals between 18 and 70 years of age; English speakers; DSM-5 criteria for CUD, MUD or OUD.	MAIA, EGNG, DTS, PROMIS-D, PROMIS-A, BPAS, SHAPS, MCQ, PSQI	TreeNet; CART
Burgess-Hull et al. (2023)	Opioid	Adherence	CS	2, 742 ind. w/ 40, 005 rec.	USA	Patients seeking OUD treatment; $40.84\pm 11.02$ years; DSM-V for OUD.	Demographic; urinalysis; attendance	LR; LR with ridge penalization; XGB
Baucum et al.(2023)	Cocaine; alcohol; cannabis; heroine; stimulants; polys.; opioid; meth	Adherence, treatment prescription	L	6, 171, 791 rec.	USA*	Admissions focusing on SUD rehabilitation; $\ge 12$ years; psychiatric evaluation for SUD	Demographic; clinical; socioeconomic; substance use; medical history	RL, MDP, RF, GBDTs, NN, LR
Koban et al. (2023)	Cocaine; alcohol; nicotine	Craving severity	CS	99 ind. w/ 469 images	USA	Participants from five previous cohorts; 34.12 mean age; DSM-IV for SUD.	fMRI;	LASSO-PCR, GLM
Shrestha et al. (2023)	Polysubs.	Craving severity	CS	60 ind.	USA	Individuals from outpatient SUD treatment programs; $\ge 18$ years; Psychiatric evaluation for SUD.	Wearable sensor GV4 features	Unclear; includes Gaussian SVM
Roberts et al. (2022)	Alcohol	Relapse	CS	1,383 ind.	USA	Individuals seeking treatment at 11 centers (COMBINE); $\ge 18$ years; DSM-IV criteria for AUD	Clinical; several different questionnaires ; substance use; medical history; biomarkers	RF
Gottlieb et al. (2022)	Opioid	Adherence	L	1, 406 ind.	USA	Patients under treatment in a SUD recovery program for more than 90/120 days, unclear diagnostic criteria.	Demographic; clinical; substance use; criminal activity; behavioral/mental health	LR, RBF SVM, RF, AdaBoost, GentleBoost, LogitBoost, RobustBoost, totally corrective boosting.
Kang et al. (2022)	Heroine	Identify SUD	CS	125 ind.	China	Male individuals; $46.09\pm 8.3$ years; DSM-V criteria for HUD.	Clinical; questionnaires; behavioral tasks	SVM
Annis et al. (2022)	Alcohol; nicotine; opioid	Identify SUD	CS	345, 728 rec.	USA	ED recs. from EHRs of a large university health system; age: $49.50\pm 21.16$ years; ICD-10 and ICD-9 codes for OUD.	Demographic; clinical; medical history; previous OUD diagnosis	LR; XGBoost
Burgess-Hull et al. (2022)	Opioid	Subgroups	CS	211 ind.	USA	Participants in a 16-week trial to develop tools for real-time assessment of exposure/response to drugs (78% male); $43.35\pm 9.57$ years; DSM-IV for SUD.	ASI: Demographics; drug use; legal problems; physical, psychological, & social health; medication	GMM, SVM
Bailey et al. (2022)	Heroine; opioid	Adherence	L	741, 483 HUD rec. 2, 322 OUD rec.	USA*	HUD and OUD admissions; $\ge 12$ years; psychiatric evaluation	Demographic; clinical; socioeconomic; substance use; medical history; diagnosis	RF, CNN
Davis et al. (2022)	Stimulants; Opioid	Relapse	CS	10, 683 ind.	USA#	Individuals on treatment with completed baseline and follow-up assessments at 3, 6, and 12 months; $\ge 12$ years, 26.9% $<18$ years; DSM-IV or V for OUD.	Demographic; clinical; geographic	RF; LASSO
Davis et al. (2021)	Opioid	Relapse	CS	1126 ind.	USA#	Individuals entering treatment; $\ge 12$ years, 26.8% 18 years; DSM-IV criteria for OUD.	Demographic; socioeconomic; clinical; substance use; medical history; psychological and behavioral; recovery and support environment	RSF; Regularized Cox Reg.
Costello et al. (2021)	Alcohol; cannabis; nicotine; stimulants; opioid; sedatives	Relapse	L	254 ind.	Canada	Individuals completing inpatient treatment with follow-ups at 1, 3, 6, or 12 months; $45.6\pm 11.2$ for mid/low involvement and $44.4\pm 10.9$ high involvement; psychiatric evaluation for SUD.	Demographic; socioeconomic; substance use; criminal activity; motivation; social support; wellbeing assessment; occupational performance; legal issues; program assessment	Multivariate LR; RF; BART
Cavicchioli et al. (2021)	Cocaine; alcohol; polys.; opioid; amphetamines; benzodiazepines	Adherence and relapse	CS	275 ind.	Italy	Individuals seeking treatment; $47.39\pm 9.15$ years; DSM-IV-TR or DSM-V for AUD	Demographic; urinalysis; mental health disorder; ASI domain scores; DERS scores; medical history	Penalized reg. combined with a nested cross-validation procedure
Nasir et al. (2021)	Alcohol; polys.; opioid	Adherence	CS	226, 940 rec.	USA*	New England residents admissions; $\ge 12$ years; psychiatric evaluation	Demographic; clinical; socioeconomic; questionnaires; length of stay	ANN, RF, LR, XGB
Jing et al. (2020)	Polys.	Identify SUD	CS	754 ind.	USA	Cohort initiating at 10-12-year-old of children of SUD parents, 75.6% EA, 21.2% AA (CEDAR); SCID-III criteria for SUD	Clinical; questionnaires; socioeconomic; psychosocial and environmental characteristics	RF; SVM; nearest neighbors; NB; MLP; LR; Adaboost
Cox et al. (2020)	Cocaine; cannabis; heroine; nicotine; opioid	Cessation	CS	4, 941 ind.	USA	Individuals recruited for genetic studies of SUD at various universities (68.3% EA; 31.7% AA); $38.77\pm 9.60$ years; DSM-V criteria for SUD	Demographic; medical history; substance use; psychiatric disorders	LASSO; SVM; RF; DNN
Morel et al. (2020)	Alcohol; polys.	Readmission	CS	65, 426 ind. w/ 97, 688 rec.	USA	Individuals with continuous insurance from multiple centers; 11-64 years; ICD-9 criteria for M/SUD	Demographic; medical history; diagnosis	XGB; GLMNet
Symons et al. (2020)	Alcohol	Adherence	CS	1, 236 ind.	Australia	Individuals from a metropolitan public hospital; $39.80\pm 11.11$ years; DSM-IV criteria for AUD.	Demographic; family history; SUD severity	RF, BayesNe, NB, SPegasos, RBFN, SMO, REPTree, DTNB, FURIA, Decision table, LR
Panlilio et al (2020)	Cocaine; opioid	Subgroups	CS	426 ind.	USA	Methadone-maintained participants assigned to contingency-management or control groups from three cohorts; no age information; DSM-IV criteria	Clinical; urinalysis	Multinomial LR, K-means, hierarchical clustering.
Suchting et al. (2019)	Cocaine	SUD severity	CS	772 ind.	USA	Individuals who took part in a two- to three-day evaluation, 75% male, 69% African-American; $44.1\pm 8.9$ years; SCID-IV for CUD.	ASI; BD-II; SCID-IV ratings	GAM, GLM
Yip et al. (2019)	Cocaine	Relapse	CS	53 ind.	USA	Participants recruited from a trial on behavioral therapy plus galantamine/placebo; DSM-IV criteria for CUD (73.6% male).	fMRI; demographic; socioeconomic; clinical; substance use	CPM MODEL
Steele et al (2018)	Cocaine; heroine; stimulants	Adherence	CS	139 ind.	USA	Incarcerated treatment-seeking individuals (36% male); $34.00\pm 7.97$ years; unclear diagnostic criteria	fMRI; demographic; clinical; socioeconomic; substance use; medical history; diagnosis	SVM
Acion et al (2017)	Coccaine; alcohol; cannabis; stimulants; polys.; opioid; hallucinogenic	Adherence	CS	99, 013 rec.	USA*	Hispanic/Latino individuals in hospitalized SUD treatment with no prior treatment history; aged $\ge 18$; psychiatric evaluation for SUD.	Demographic; socioeconomic; clinical; substance use; length of stay	LR; penalized reg.; RF; ANN; super learning (ensemble).
Steele et al. (2014)	Cocaine; heroine; stimulants	Adherence	CS	144 ind.	USA	Incarcerated treatment-seeking individuals, 65% hispanic; $35.48\pm 8.47$ years; unclear diagnostic criteria	Demographics; EEG features; questionnaires; psychiatric disorders	SVM

**Notes**: *Polys*. polysubstance, *CUD* cocaine use disorder, *OUD* opioid use disorder, *AUD* alcohol use disorder, *HUD* heroin use disorder, *MUD* marijuana use disorder, *DSM* diagnostic and statistical manual of mental disorders, *SCID* structured clinical interview for DSM, *CS* cross-sectional, *L* longitudinal, *Ind* individuals, *Rec* records, *EA* European American, *AA* African American, *LOS* length of stay, *LR* logistic regression, *RL* reinforcement learning, *MDP* Markov decision process, *RF* random forest, *RSF* random survival forest, *GBDTs* gradient boosting decision trees, *RBF* radial basis function, *NN* neural networks, *SVM* support vector machine, *BART* Bayesian additive regression trees, *GAM* generalized additive models, *GLM* generalized linear models, *GLMM* generalized linear mixed model, *NB*: Naive Bayes, *DTNB* Decision Table/Naive Bayes, *GMM* growth mixture models, *CPM* connectome-based predictive modelling, *DNN* deep neural network, *ANN* artificial neural network, *CNN* convolutional neural network, *XGB* XGBoost, * TEDS-D dataset, # GAIN dataset

The sample size varied from 53 to 65,426, considering the studies that analyzed individual patients. This comprised 195, 839 individuals if there were no overlaps between datasets. The studies that used treatment records as primary data varied from 2322 to 6, 171, 791 admissions. The TEDS-D, used by 16% of the reviewed studies, is an example of a public dataset organized through treatment records, considering each admission as a different instance. This creates an inherent risk of redundancy, as this approach may inadvertently consider the same patient multiple times as different ones if they have undergone multiple admissions.

Input features across studies varied widely, with 17 models incorporating demographic and socioeconomic information like age and gender; 19 studies included clinical data and medical history, detailing diagnoses and treatment histories; substance use patterns were noted in 11 studies; psychological and behavioral assessments appeared in 9 studies; and EEG, fMRI data, and biological markers like urinalysis were used in 6 studies each. Treatment-related features were featured in 6 studies. Diagnostic criteria varied significantly, with DSM leading as the most used criteria in 16 studies (DSM-IV: 9; DSM - V: 4; Both: 2), followed by psychiatric evaluation in 6 studies, ICD-9 in two studies, and SCID-I, SCID-III, and SCID-IV in one study each. Three studies did not specify the diagnostic criteria used.

**Fig. 2:**
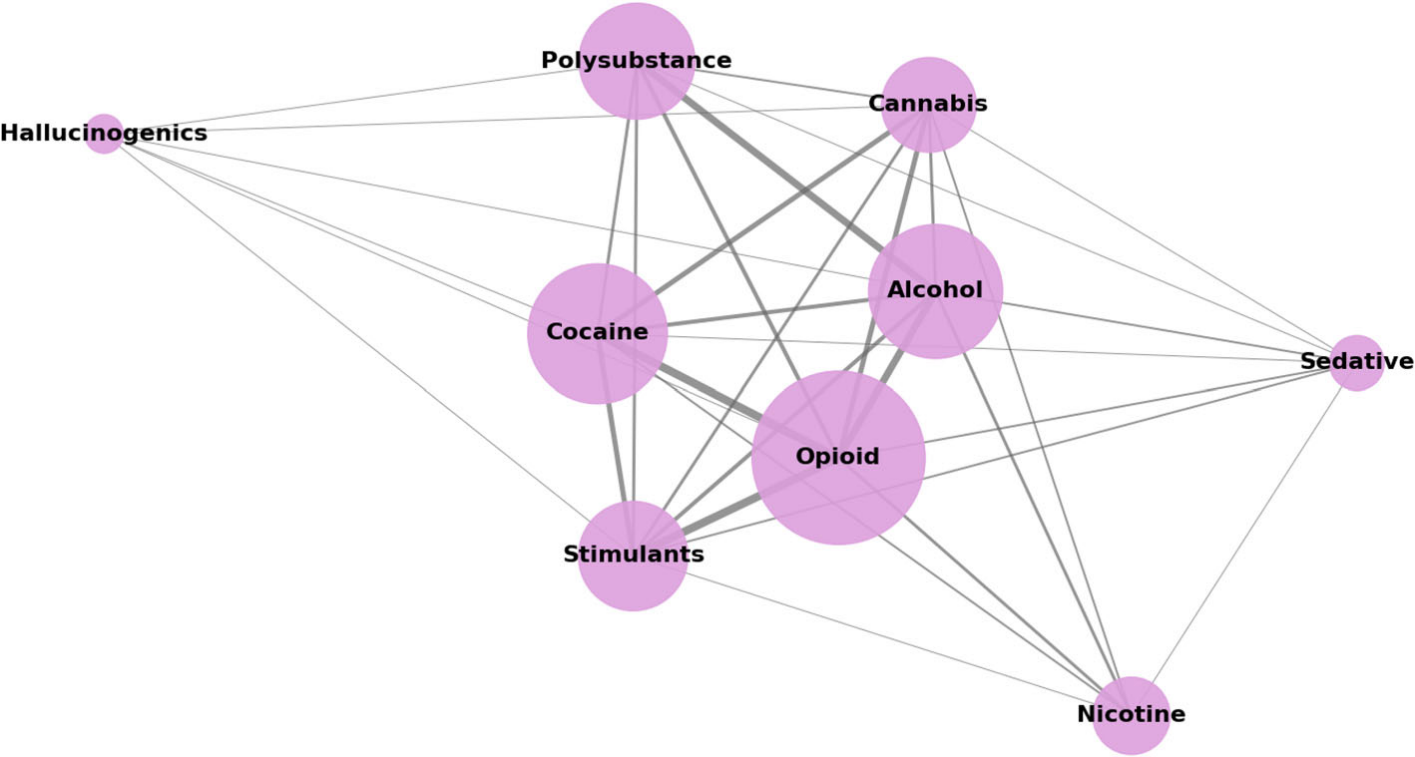
Network diagram visualizing the primary substances examined across the surveyed studies. Each node represents a class of substance, and the diameter of each node is scaled according to the frequency of studies investigating disorders related to that specific substance, thereby reflecting its prevalence in the research. Nodes are positioned closer together when substances are frequently studied in combination, indicating higher co-occurrence within the same studies. The thickness of the edges between nodes represents the strength of this co-occurrence, with thicker lines indicating a higher frequency of joint investigation. Figure obtained using networkx and matplotlib package on python

Figure 2 presents a network diagram showcasing the primary substances investigated within the included studies. The analysis revealed a diverse scope of substances with a pronounced focus on opioids. This emphasis predominantly reflects the research priorities and the ongoing opioid crisis within the US, where the overwhelming majority of the samples from the studies originate, and the substantial funding directed toward opioid research. In addition to opioids, alcohol, and cocaine were also significantly represented, underscoring their global prevalence and impact on contemporary health and addiction research concerning SUD. Polysubstance was also reported in several studies, describing the prevalence of multiple drug use without a specific preference.

Figure S1 (Supplementary Materials) presents the main ML models used as well as the most commonly used input features.

**Fig. 3:**
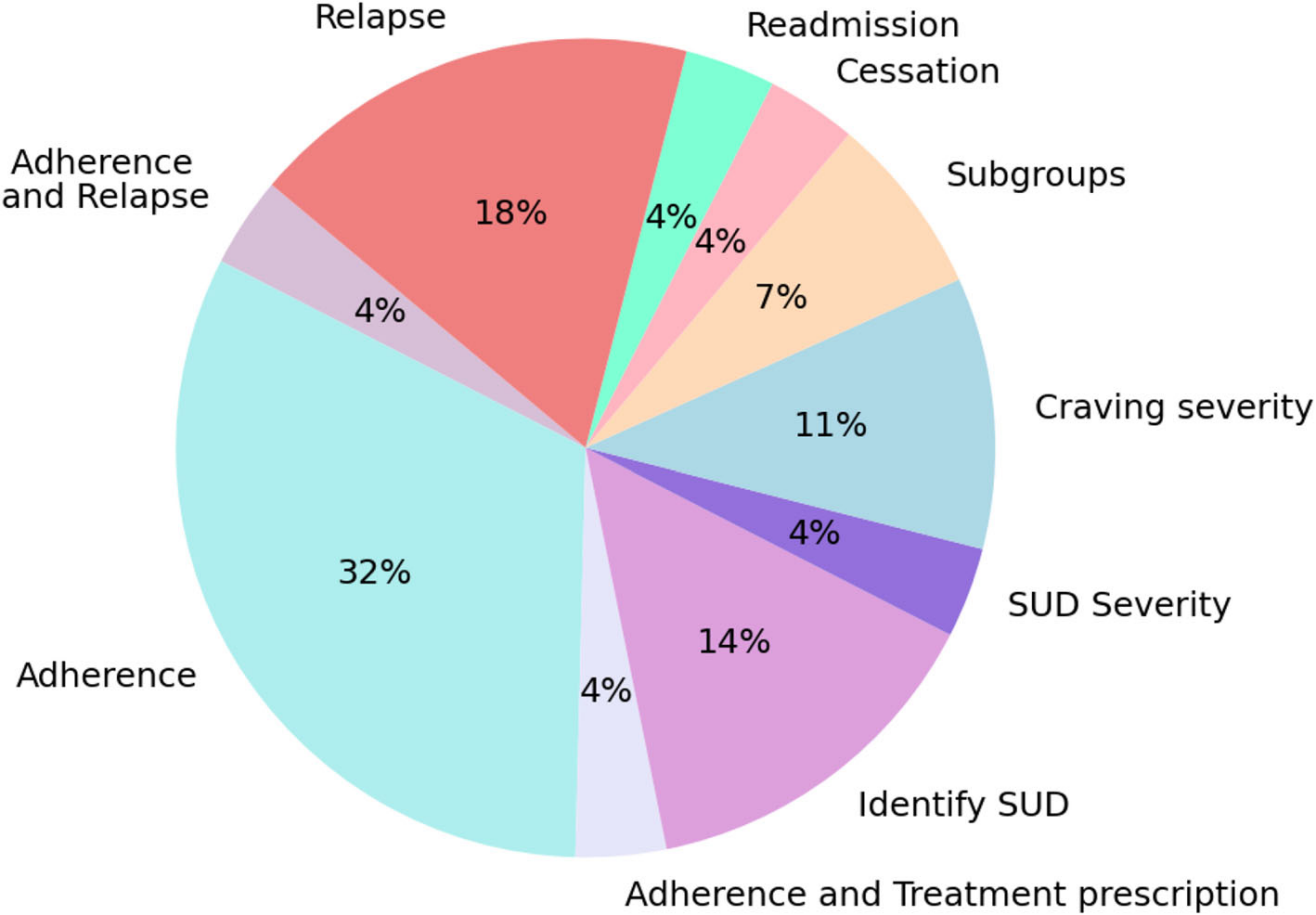
Proportion of clinical outcomes predicted with ML in the reviewed studies

Figure 3 shows the analyzed outcomes of the reviewed studies. A notable trend was the predominant focus on predicting treatment adherence and relapse, crucial indicators of long-term treatment success in SUD management.

According to the CHARMS and MI-CLAIM evaluations (Table S3, Supplementary Materials), most studies assessed had several shortcomings, from lack of clarity on the dates the dataset had been collected on, lack of information of feature selection and importance, percentage of participants with missing values, characteristics of both the training and test datasets, reporting of consistent metrics, discussion of robustness and reliability of the models, and reproducibility.

## Discussion

This systematic review synthesized the findings of 28 publications that explored the application of ML algorithms to the prediction and analysis of treatment outcomes in SUDs. The increasing number of studies published in this area underscores a growing recognition of the potential of ML models within the addiction research community. This emerging movement may offer novel insights that can potentially reshape our understanding of effective SUD interventions. However, several major problems were found, such as inconsistencies in reporting, particularly regarding the data analyzed, in model performance, in data leakage prevention (or lack thereof), in code transparency, and in dataset characteristics. These gaps highlight the need for standardization and rigor in ML applications within SUD research to ensure reproducibility and clinical relevance.

Our findings are in accordance with the broader evidence identified in recent literature, which delineates the emerging integration of ML within various health domains (Andaur Navarro et al., 2023; Andaur Navarro et al., 2023, 2022; Dhiman et al., 2022, 2023; Collins et al., 2014; Farimani et al., 2024). This collective body of work presents a confluence of promising results as well as shortfalls in methodological rigor. Notably, in the addiction research field, similar explorations (Chhetri et al., 2023; Mak et al., 2019) parallel our observations, albeit without a specific focus on methodological critique and clinical outcome orientation that our review prioritizes. To present our findings and guide the readers to points of interest, we break down the revised studies according to the analyzed clinical outcome. For studies adhering to best practices in ML and medical research, we highlight their significant contributions and insights. Conversely, for studies demonstrating methodological biases and oversights, we scrutinize their implications on the findings and propose viable methodological alternatives.

### Outcomes

ML was used to predict several SUD-related clinical outcomes, including identifying SUD, assessing treatment adherence, evaluating severity, identifying disease subtypes or trajectories, and predicting relapse, cessation, and readmission. The distribution of these outcomes across the reviewed studies is synthesized in Fig. 3.

#### Identifying SUD

An SUD diagnosis relies on the presence of specific symptoms indicative of substance misuse,  American Psychiatric Association (2022) (ICD) World Health Organization (2024). The number of symptoms determines the severity (mild, moderate, or severe). The diversity in SUD symptomatology, influenced by the broad spectrum of diagnostic criteria, is a challenge reflected in predictive modeling. Accurate representation of this clinical variability in the data used to train ML models is critical for their effectiveness. Four studies in this review shed light on ML’s ability to distinguish individuals at high risk from those unlikely to develop such disorders. Annis et al. (2022) and Kang et al. (2022) sought to differentiate healthy controls from individuals with SUD, whereas Jing et al. (2020) aimed to forecast SUD risk in offspring of SUD-affected adults. Additionally, Houghton et al. (2023) sought to classify SUD individuals as having significant comorbid PTSD symptoms based on symptoms of anxiety and depression, as well as perseverative/intrusive thought patterns, low tolerance of aversive body sensations, effectively differentiating PTSD-comorbid patients from those with only SUD.

Annis et al. (2022) used a distinct definition of opioid use disorder (OUD) through multiple ICD codes, which includes other conditions, such as specific adverse effects of opioids and opioid poisoning. While this assumption is clinically acceptable for ML model training, some limitations should be noted. The reliance on treatment records is susceptible to data leakage through repeated patient admissions. Furthermore, the dataset’s imbalance, with “no OUD” cases vastly outnumbering “OUD” cases, suggests that model accuracy may inadvertently reflect dataset skew rather than genuine predictive capability. The model’s performance corroborates this: while accuracy is close to 99% in all experiments, the area under the curve (AUC) never exceeds 0.5. Another empirical concern, given the chronic nature of SUD diagnosis (American Psychiatric Association, 2022), is the inclusion of “previous diagnosis of OUD” as a predictive feature, as the model mainly relied on the previous diagnosis instead of making its own prediction.

Kang et al. (2022)’s model demonstrates adeptness in distinguishing between healthy controls (HC) and individuals with OUD. However, the contexts for the OUD and HC groups should be highlighted. OUD individuals were recruited in a compulsory psychiatric facility, which would filter for more impulsive individuals, while HC were recruited from the local community. It is possible that the model learned to differentiate these two contexts instead of identifying the presence of SUD. This is further corroborated by the model’s reliance on impulsivity-related features as predictors, which are absent from SUD diagnostic criteria, calling into question the model’s clinical relevance. This would also explain how they reached an abnormal AUC of 0.99 and 96% accuracy.

Jing et al. (2020) stand out as one of the few included longitudinal studies, monitoring a cohort of children from families affected by SUDs from infancy to adulthood, with the objective of forecasting the development of SUD. The model’s AUC ranges from 0.7 to 0.8, increasing with age, which indicates a reasonable accuracy in predicting SUD onset and could be used to identify high-risk individuals for prior intervention. Despite their disconnection from traditional SUD risk, their findings suggest that early childhood behaviors (such as swearing) might provide a deeper understanding of developmental psychopathology and may offer early signs of developmental trajectories that predispose to SUD.

#### Treatment Adherence and Dropout

The chronic nature of many disorders requires prolonged engagement with therapeutic interventions to ensure effective treatment and care continuity. Consequently, treatment adherence/completion is crucial for effective treatment of SUDs. Due to their design, treatment modalities, and limitations, the adherence criteria vary throughout SUD research, including program completion, dropout rates, and appointment attendance. Nine studies (Acion et al., 2017; Bailey & DeFulio, 2022; Burgess-Hull et al., 2023; Eddie et al., 2024; Gottlieb et al., 2022; Steele et al., 2018, 2014; Symons et al., 2020; Nasir et al., 2021) focused exclusively on adherence metrics, while two others also included secondary analyses: relapse (Cavicchioli et al., 2021) and treatment prescription (Baucum et al., 2023). Table 2 sheds light on the input features and models employed, which range from traditional logistic regression (LR) to more advanced algorithms like XGBoost and convolutional neural networks (CNN).

**Table 2:** Characteristics of the studies focused on predicting treatment adherence

Ref.	Input features	Adherence criteria	Best model	Metrics of best model
Eddie et al. (2024)	Demographic; psychosocial; treatment-specific	Attended at least 3 sessions	GLMM	AUC 0.84
Baucum et al. (2023)	Demographic; clinical; socioeconomic; substance use; medical history	Treatment completion*	XGBoost	AUC 0.80
Burgess-Hull et al. (2023)	Demographic; urinalysis; attendance so far	Attendance at next appointment	Random forest	AUC 0.87
Bailey and DeFulio (2022)	Demographic; clinical; socioeconomic; substance use; medical history; diagnosis	Treatment completion*	CNN	AUC 0.49
Gottlieb et al. (2022)	Demographic; clinical; substance use; criminal activity; behavioral/mental health	Treatment completion	Random forest	Sensitivity 0.81; Specificity 0.65
Cavicchioli et al. (2021)	Demographic; urinalysis; mental health disorder; ASI domain scores; DERS scores; Medical history	Missed at maximum 3 appointments	LR	AUC 0.76
Nasir et al. (2021)	Demographic; clinical; socioeconomic; questionnaires; length of stay	Treatment completion*	XGBoost	AUC 0.89
Symons et al. (2020)	Demographic; family history; SUD severity	Treatment completion	RBFN and LR	AUC 0.64
Steele et al. (2018)	Demographic; clinical; socioeconomic; substance use; medical history; diagnosis	Treatment completion	SVM	Sensitivity 0.82; Specificity 0.78
Acion et al. (2017)	Demographic; socioeconomic; clinical; substance use; length of stay	Treatment completion*	Super learning (ensemble)	AUC 0.82
Steele et al. (2014)	Demographics; EEG features; questionnaires; psychiatric disorders	Treatment completion	SVM	Sensitivity 0.83; Specificity 0.78

**Notes:** *GLMM* generalized linear mixed model, *CNN* convolutional neural network, *LR* logistic regression, *RBFN* radial basis function network, *SVM* support vector machine. Treatment completion*: used TEDS-D treatment completion criteria

Four studies utilized the TEDS-D dataset to train and evaluate models to predict treatment completion. They used similar input features, and three of them reached $>0.8$ AUC. Yet, methodological oversights should be considered.

The study with a lower AUC (Bailey & DeFulio, 2022) investigated the use of transfer learning. They trained a deep learning model on an OUD dataset and then finetuned (continued training) it on another OUD dataset. This is a promising approach that, if successful, could enhance SUD-related predictions. However, the execution faced critical limitations, particularly in the premature cessation of the model’s training phase, with no proper justification, and its evaluation. While the accuracy is 76%, this derives from the dataset imbalance. Specifically, the model exhibited a pronounced bias toward predicting treatment completion, correctly identifying only 37 instances of Treatment Failure against a ground truth of 310 out of 1, 161 patients.

The TEDS-D dataset variable “length of stay” (LOS) indicates the number of days an individual stayed in treatment. LOS is directly related to the treatment outcome, as low values indicate dropout or other reasons for treatment termination. However, it is unavailable for individuals entering treatment. Accordingly, LOS should not be used to predict treatment adherence. Still, two studies (Acion et al., 2017; Nasir et al., 2021) used it as an input feature to the ML model in order to predict treatment completion. Unsurprisingly, LOS was the most important variable in both study models, which unfortunately skews their whole analysis. Their model high AUC is problematic when considered representative of a clinical setting. Baucum et al. (2023) aim to predict SUD treatment completion and assign treatment modality using both ML and reinforcement learning. They reached an AUC of 0.80 for predicting treatment completion and 0.87 for predicting the optimal treatment plan as determined by each individual’s LOS. Showing no primary limitations, it is evidence that ML might be used to identify individuals with a high risk of not completing treatment.

An additional six studies used other datasets to predict adherence-related outcomes: Symons et al. (2020) juxtaposed the predictive accuracies between ML models and seasoned clinical psychologists on successful alcohol use disorder (AUD) treatment estimation. They used several models for this comparison and adequately only provided information available at treatment initiation to both psychologists and ML models. ML models, especially LR and radial basis function networks, provided more accurate predictions overall. Burgess-Hull et al. (2023)’s approach integrates longitudinal urinalysis and clinical data to predict the next appointment attendance of OUD patients. Their strong AUC of 0.87 emphasizes real-time data integration up to the last appointment and showcases the methodological diversity in harnessing ML for insights into SUD applications. Cavicchioli et al. (2021)’s study aims to predict dropout or relapse in individuals with a primary diagnosis of AUD. They used a regularized LR model, achieving an AUC of 0.76 on predicting drop-out. Higher ASI alcohol scores were the most important feature for prediction.

Steele et al. (2014)’s study uses EEG features from a small cohort of 144 incarcerated individuals to identify patients who discontinued their SUD treatment. EEG features were collected during a Go/NoGo task — specifically stimulus-locked, response-locked, and Event-Related Potentials. They achieved high performance with P2, ERN/NE, and Pe amplitude (brain processes information, responds to errors, and attention and cognitive control) as the strongest predictors for treatment discontinuation, as hypothesized by the authors. Their more recent study (Steele et al., 2018) used several clinical features, including fMRI ones, in another Go/NoGo task. The high performance of both study’s models showcases the relevance of EEG and fMRI features in the context of adherence. Gottlieb et al. (2022) aimed to identify individuals with OUD at higher risk of dropout after 90 and 120 days. Despite imputating features with excessive missing data (50%) and the lack of reports of AUC values, the results were promising, reaching a high sensitivity and specificity. The most important feature of their model prediction was the patients’ quality of life. However, the lack of detailed reporting on SUD diagnoses in these three studies (Steele et al., 2014, 2018; Gottlieb et al., 2022) is a significant methodological shortfall that should be considered in future research, as it impacts the interpretability and generalizability of their findings.

Despite the identification of significant methodological shortcomings in certain studies (Nasir et al., 2021; Acion et al., 2017; Bailey & DeFulio, 2022), which ultimately undermines the reliability of their findings, it is important to recognize that a body of work exists wherein only minor discrepancies or none at all are observed. These studies offer substantial evidence to support the efficacy of predictive modeling in the context of SUD treatment adherence. Accurately identifying patients at heightened risk of dropout provides a valuable foundation for developing targeted interventions and support mechanisms aimed at bolstering adherence rates among SUD patients.

#### Relapse, Cessation, and Readmission

In the realm of SUDs, terms such as relapse, cessation, and readmission present complex and important phenomena of recovery and treatment. Relapse, clinically defined as a return to substance use after a period of abstinence, underscores the chronic nature of SUD (National Institute on Drug Abuse (NIDA), 2023). It highlights the challenges in maintaining long-term recovery in the face of potential stressors. Cessation, or the successful discontinuation of substance use, represents a critical goal of SUD treatment. Readmission reflects recurrent hospitalizations or treatment enrollments, often referred to as the “revolving door phenomenon.” It points toward the need for more effective or sustained intervention strategies. Predicting these outcomes has significant clinical implications. It enables tailoring treatment plans to individual patient needs, potentially improving care continuity and outcome efficacy. Such predictive efforts can inform the allocation of healthcare resources, guiding timely and appropriate interventions for patients at heightened risk. From the eight papers that fall under this category, five predicted relapse  (Cavicchioli et al., 2021; Davis et al., 2021, 2022; Roberts et al., 2022; Costello et al., 2021) while the others predicted treatment readmission (Morel et al., 2020), abstinence (Yip et al., 2019), and cessation of use (Cox et al., 2020).

Aside from dropout, Cavicchioli et al. (2021) also used regularized LR to predict relapse, but their AUC of 0.51 shows their model was unreliable. Davis et al. (2021) aim to identify relevant features for predicting relapse independently for women and men. While the authors extensively discuss their findings according to the features with the highest impact on the model outcome, the model’s performance is alarmingly poor. The accuracy reported is 56.8% for women and 62.3% for men, which is only marginally better than 50% accuracy of random choice. This causes any subsequent analyses of the most important features of the model decision meaningless, given that the model is probably relying on random features unrelated to the outcome. Their next study (Davis et al., 2022) explores the prediction of post-treatment opioid and/or psychostimulant use. The choice of hazard ratio and concordance index metrics for evaluation is unusual and limits the interpretability and applicability of their model. Their focus lingers on analyzing the most important features while giving little importance to the model performance.

 Roberts et al. (2022) developed models using routinely collected clinical data to predict heavy drinking in patients completing a structured outpatient treatment program — the COMBINE trial (Anton et al., 2006). This study stands out for its innovative application of the leave-site-out (LSO) validation method alongside traditional 10-fold cross-validation. Employing LSO effectively mimicked a real-world scenario in which predictive models should perform well not only in institutions that provide the data used to train them but also on data from other sources. The AUC of 0.71, 0.70, and 0.84 to predict heavy drinking during the first month, final month, and between sessions, respectively, showcases the generalizability and applicability of their model. Costello et al. (2021) use conventional and ML propensity score-based methods to examine the effectiveness of 12-step group involvement in reducing relapse following SUD treatment. They demonstrated that high involvement in the group indeed reduces the likelihood of relapse.

Morel et al. (2020) analyzed readmission within 30 days as a key outcome for both mental and substance use disorders. The training and testing datasets were appropriately divided and described. They reached an AUC of 0.74 with XGBoost, showcasing the potential to identify patients in need of extra care for effective treatment. The most important predictor was length of stay, but several other features also significantly impacted the model prediction. Yip et al. (2019) aim to identify a brain-based predictor of cocaine abstinence, using fMRI and clinical information. They used connectome-based predictive modeling and were able to achieve an accuracy of 0.71. However, the dataset comprises only 53 incarcerated individuals and cannot be considered representative of a real-world scenario. Moreover, the study does not state the diagnosis criteria used to select the participants.

Cox et al. (2020) study aimed to identify the different factors associated with drug use cessation in African-(AA) and European-American (EA) samples. They defined cessation as having last used opioids $>12$ months before the interview and non-cessation as $<6$ months before the interview, with intermediate data excluded from the analysis. This practice is an oversight that leads to artificially-improved performance. A more reliable approach would be to use an intermediate cut-off point for cessation. Still, this cut-off dropped only a few instances, and their SVM model reached an accuracy of 0.75 and 0.79 on AA and EA, respectively. They found that for AA, drug-related predictors were mostly cocaine-related, as in EA, stimulants were also relevant.

#### Severity

The assessment of SUD severity is substantial for effective treatment approaches to individual patients’ needs. Clinically, the severity of SUD is quantified based on diagnostic criteria outlined in the DSM (American Psychiatric Association, 2022) and the ICD (World Health Organization, 2024). Similarly, craving severity is inherently tied to the individual’s difficulty in managing or reducing substance use despite a desire to do so. The heterogeneity in conceptualizing and measuring SUD severity presents challenges for both clinical practice and applying ML in SUD treatment research. Across the reviewed studies, varying approaches to quantifying severity are evident, with three studies concentrating on the severity of cravings  (Heberle et al., 2024; Shrestha et al., 2023; Koban et al., 2023) and one on SUD severity (Suchting et al., 2019). This diversity underscores the complexity of SUD as a clinical condition and highlights the need for precise and adaptable metrics in ML applications.

Shrestha et al. (2023) employ a wrist-worn sensor in a relatively small cohort of 60 individuals who own smartphones, analyzing craving and stress. Despite the innovative approach and the several performed analyses, data from the same participants were included across different cross-validation folds, indicating data leakage. This artificially improved performance and the absence of the ML model description raise doubts regarding the study’s applicability.

 Koban et al. (2023) aim to differentiate drug users from non-users through a proposed neuromarker from fMRI features, using LASSO-PCR and GLM models. Using self-reported craving as the outcome, they demonstrated their approach reaches an AUC of 0.91 on differentiating high and low craving. Subsequent analyses showed that food cues are not enough to differentiate between drug users and non-users (AUC 0.4). However, drug cues were effective (AUC 0.76), and using data from both food and drug cues increased performance even further (AUC 0.87).

Despite claiming to analyze SUD severity, the primary analysis of Suchting et al. (2019) study is the proportion of cocaine use days in the last 30 days. The ML model predicts whether the individual used it in the last 30 days. A secondary investigation analyses cocaine abuse and the severity of this diagnosis through comorbidities, exploring depression symptoms as a predictor for SUD severity. It brought insights into how depression and SUD are correlated, and the authors consider that interventions designed to target cocaine use might benefit from particular management of the patient’s emotional volatility and constricted perception of the future.

#### Clustering and subtypes

Clustering is an unsupervised ML technique that arranges patients into groups (called clusters) based on patterns in the data. Identifying groups within SUD patients may uncover a nuanced understanding of the disease, enabling more targeted and effective treatment approaches based on patient profiles. Our review encompasses two different clustering approaches in the SUD context (Burgess-Hull et al., 2022; Panlilio et al., 2020).

Burgess-Hull et al. (2022) employed growth mixture models to discern distinct craving trajectories among opioid-use patients undergoing treatment with buprenorphine or methadone. They identified four unique patterns and corresponding sample sizes: rapidly declining craving — 7.6%; high and increasing craving (HIC) — 10.9%; low craving(LC) — 73%; and increasing and decreasing craving — 8.5%. The considerable size difference between these groups, particularly with the LC trajectory comprising most of the sample, underscores the diverse patient experiences and responses to treatment. Given the potential necessity for augmented therapeutic strategies within the HIC group, the study also explored supervised prediction methodologies using a subset of baseline ASI variables and one, two, or three weeks of in-treatment data. The best model used two weeks of in-treatment data and reached a praiseworthy performance. Even though not all HIC patients were correctly identified (PPV of 0.5), the model’s high specificity (NPV of 0.97) emphasizes the model’s potential for clinical applications.

Panlilio et al. (2020) employed hierarchical and *k*-means clustering methodologies to urinalysis test results, aiming to uncover patterns of drug use among individuals undergoing methadone treatment for opioid and cocaine use disorder. The resultant clusters — exclusive opioid use, exclusive cocaine use, concurrent opioid and cocaine use, and varying degrees of abstinence — mirrored distinctions that could arguably be deduced without sophisticated ML techniques, raising questions about the necessity and added value of employing clustering in this context. This direct correspondence suggests a missed opportunity to explore more complex, less intuitive patterns that could offer novel insights into treatment effectiveness or patient behavior.

### Main Recommendations for ML Applications in the Field of SUD

This systematic review highlights the intersection of ML and SUD treatment outcomes, revealing a landscape replete with promise — especially in identifying high-risk patients prone to dropout and relapse — but also marked by significant methodological variability and challenges. Given the limitations encountered during this exploration, we urge the adoption of specific standards and protocols to ensure the integrity, rigor, and applicability of future ML applications in SUD treatment. This section introduces a structured set of guidelines and recommendations tailored to address the challenges unveiled through our systematic investigation.

**Model Validation**: for ML models to be truly beneficial in the SUD treatment landscape, they need to be rigorously validated in unseen data. While cross-validation is a standard step for preliminary evaluation, we advocate for including external validation using independent test datasets, using data that were not used to train or validate the models. Highlights in the reviewed studies include the leave-site-out approach employed by Roberts et al. (2022). Proper evaluation also requires proper use of context-relevant metrics and addressing biases such as data imbalance and data leakage, which will be discussed next.

**Proper reporting of Metrics**: the diversity in model evaluation metrics and lack of standardization complicate comparing and integrating findings across studies. To foster consistency, comparability, and applicability, we recommend adopting standardized evaluation metrics relevant to an SUD treatment context. While subsequent analyses may use other metrics, AUC, sensitivity, and specificity are key indicators that should be included in all ML studies that predict clinical outcomes (Tan et al., 2018). Specifically, AUC reporting is important as it provides information on the model’s performance across different thresholds. Additional metrics should also be included, as each one explores different characteristics of the model, giving the reader a better understanding of its strengths and limitations. The study by Burgess-Hull et al. (2023) is a good example of adequate reporting of several metrics. Further, we recommend making the confusion matrix available as several metrics can be computed from it.

**Data Imbalance**: it is a pervasive challenge in ML model training, where the proportion of data available for each outcome is vastly different. This imbalance can skew model predictions, favoring the majority class and undermining the model’s clinical utility, a problem encountered in several studies in this review (Annis et al., 2022; Bailey & DeFulio, 2022; Cox et al., 2020). To mitigate this problem, we advocate for implementing data augmentation techniques and/or applying balanced batch training methods. Outlier recognition algorithms, such as isolation forests, are recommended for extremely unbalanced datasets, as they perform better in these circumstances. The choice of metrics is also extremely relevant in this context: the accuracy of a model that always predicts the same outcome might be extremely high if the model is being validated in an imbalanced set, even though the model has no clinical relevance.

**Feature Selection and Importance**: feature selection is critical as features should be chosen according to the research context and the desired outcome. In order to predict a future event, we must restrict our analysis to variables that are available at the prediction timepoint. There is no reason to include variables that are only available at the outcome timepoint, as reported in some studies (Acion et al., 2017; Nasir et al., 2021). Ablation studies using different feature subsets according to their accessibility (such as questionnaires compared to fMRI data) are also recommended, as they help to understand the nuances and difficulties of implementing ML models in clinical practice.

SUD research shows a trend to identify predictors of a given outcome by analyzing the most important features for the model’s prediction. Verifying that the model is performing well in unseen data is imperative for this process. If the model performs similarly to random guessing, or if it mostly predicts the same outcome, then the important features for prediction are probably random. This analysis should also be performed with unseen data so that it does not end up being redundant.

**Data Leakage**: it happens when the information used to train an algorithm includes unexpected additional information about the subject it’s evaluating, inflating model performance metrics, and providing an unrealistic assessment of a model’s real-world applicability. An issue we found in a few studies (Baucum et al., 2023; Annis et al., 2022; Shrestha et al., 2023; Nasir et al., 2021; Bailey & DeFulio, 2022; Acion et al., 2017) is that some of the public datasets are based on treatment records rather than individuals. Since readmission is common on SUD, multiple entries can be considered for the same patient, which might inflate model performance on the test set. To prevent data leakage, strict data partitioning protocols must be employed, ensuring that no information from the test set — even different records from the same individuals — is available during the model training phase.

**Transparency**: few of the studies presented in this review have shared the codes used to create their models (Bailey & DeFulio, 2022; Cox et al., 2020; Koban et al., 2023; Roberts et al., 2022), which is necessary for replicating their finding. Besides, there was a lack of overall transparency on input features, and the validation process for the models was present in the vast majority of studies. Enhancing code and dataset transparency is important for allowing replication studies and accelerating innovation. Whenever possible, researchers should share their code and anonymized datasets.

## Limitations

This systematic review, while comprehensive in its approach and methodology, is subject to some limitations. Our review was dedicated to exploring the use of machine learning in predicting and analyzing SUD treatment outcomes, which inherently limited our scope to certain types of studies. Specifically, while we sought to encompass a broad spectrum of ML applications within this domain, we intentionally excluded some neurobiological studies that did not explicitly address treatment outcomes. The included studies’ heterogeneity in methodologies, treatment focuses, and reported outcomes posed challenges in synthesizing a unified narrative. Additionally, differences in diagnostic criteria such as DSM and ICD can complicate the comparison of study outcomes, potentially affecting the generalizability and interpretability of ML model predictions in SUD research. This varied and often incompatible landscape highlights the urgent need for standardized reporting and methodological guidelines as well as the emergent nature of ML applications in SUD research. Accordingly, we discussed recommendations for future studies to improve research integrity, reproducibility, and transparency.

Moreover, a significant limitation of our review lies in the data reporting and model evaluation practices observed across the included as well as the sample populations of the datasets, given most are from the US and overlap (U.S. Department of Health and Human Services, Substance Abuse and Mental Health Services Administration, Office of Applied Studies, 2019). A major concern is the reliance on treatment records which are frequently reused across multiple studies. This reuse can introduce redundancy, leading to potential biases in machine learning models that predict SUD outcomes. The use of these overlapping datasets restricts the diversity of clinical outcomes that can be explored, as the variables are often limited to those available within the datasets. For instance, many datasets predominantly include variables related to treatment completion and attendance, while nuanced outcomes related to patient psychological states or longer-term recovery are seldom reported.

## Conclusion

This systematic review has brought light to the potential of ML algorithms in predicting and analyzing treatment outcomes within SUDs. Through a critical examination of 25 studies, we identified promising capabilities of ML models in forecasting key aspects such as treatment adherence, SUD identification, relapse, and readmission for several different substances. Innovative approaches included using fMRI, mobile sensors, and urinalysis data, as well as clustering methods to further our understanding of SUD. Despite these promising insights, our investigation underscores the critical necessity for greater methodological rigor and transparency in applying ML within the SUD treatment landscape. Given these limitations, we provided recommendations and guidelines for replicable and applied research, focusing on the suitable choice of metrics and features as well as adequate validation of models. Subsequent research may significantly impact the treatment and management of SUDs by addressing current limitations, following good practices, and centering on clinically pertinent inquiries. As shown by several reviewed studies, we believe that ML has the potential to revolutionize the SUD field, especially concerning the identification of high-risk patients for dropout and relapse.

## Supplementary Information

Below is the link to the electronic supplementary material.

Supplementary file 1 (pdf 252 KB)
