# Supplementary material for: Craving for a Robust Methodology: A Systematic Review of Machine Learning Algorithms on Substance-Use Disorders Treatment Outcomes
Source: Int J Ment Health Addict. 2024 Oct 4;24(2):1090–117. doi: 10.1007/s11469-024-01403-z (PMC13139223; doi:10.1007/s11469-024-01403-z)
Supplement: Supplementary file 1 — (pdf 252 KB) [file 11469_2024_1403_MOESM1_ESM.pdf]

Blinded Manuscript Click here to view linked References

Supplementary Materials

Figure S1 presents the main ML models used as well as the most commonly used input features. While supervised models (n = 26) learn to predict outcomes based on the input data, unsupervised models (n = 2) do not have a proper outcome label to predict. Instead, they explore the data distribution to identify patterns and uncover meaningful insights.

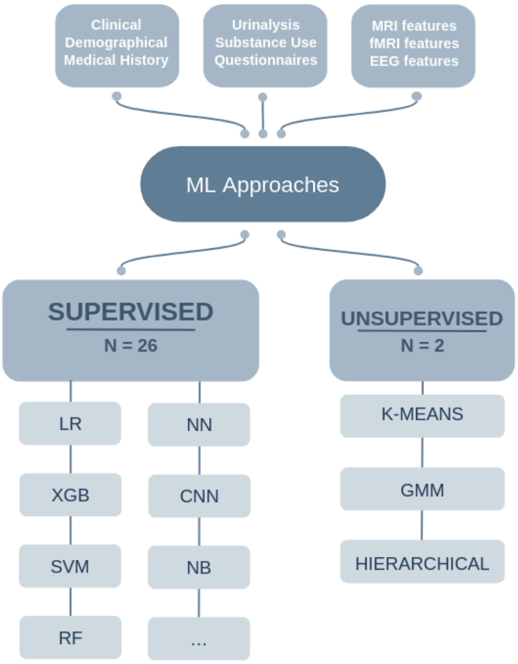

Fig. S1 Diagram presenting main input features and ML approaches used in the reviewed studies. While most studies used supervised techniques, two studies also explored unsupervised methods. LR: Logistic Regression, NN: Neural Networks, XGB: XGBoost, CNN: Convolutional Neural Network, SVM: Support Vector Machine, RF: Random Forest, NB: Naive Bayes, GMM: Growth Mixture Models.

Database and search strategy adaptations:

PubMed: (”substance-related disorder*” OR ”substance use disorder*” OR (”drug” AND ”addiction”) OR ”drug dependence” OR ”alcohol addiction” OR ”opioid addiction” OR ”cocaine addiction” OR ”methamphetamine addiction” OR ”cannabis addiction”) AND (”machine learning” OR ”deep learning” OR ”neural network” OR ”supervised learning” OR ”predictive modeling” OR ”supervised learning” OR ”support vector machine*” OR ”random forest” OR ”gradient boosting”) AND (”Readmission” OR ”Rehospitalization” OR ”Relapse” OR ”treatment outcome*” OR ”Severity”)

Web of Science: TS=(”substance-related disorder” OR ”substance use disorder” OR (”drug” AND ”addiction”) OR ”drug dependence” OR ”alcohol addiction” OR ”opioid addiction” OR ”cocaine addiction” OR ”methamphetamine addiction” OR ”cannabis addiction”) AND TS=(”machine learning” OR ”deep learning” OR ”neural network” OR ”supervised learning” OR ”predictive modeling” OR ”support vector machine” OR ”random forest” OR ”gradient boosting”) AND TS=(”Readmission” OR ”Rehospitalization” OR ”Relapse” OR ”treatment outcome” OR ”Severity”) OR ”Severity”)

Scopus: TITLE-ABS-KEY(”substance-related disorder” OR ”substance use disorder” OR ”drug AND addiction” OR ”drug dependence” OR ”alcohol addiction” OR ”opioid addiction” OR ”cocaine addiction” OR ”methamphetamine addiction” OR ”cannabis addiction”) AND TITLE-ABS-KEY(”machine learning” OR ”deep learning” OR ”neural network” OR ”supervised learning” OR ”predictive modeling” OR ”support vector machine” OR ”random forest” OR ”gradient boosting”) AND TITLE-ABS-KEY(”Readmission” OR ”Rehospitalization” OR ”Relapse” OR ”treatment outcome” OR ”Severity”)

Embase: ('substance-related disorder':ab,ti OR 'substance use disorder':ab,ti OR ('drug':ab,ti AND 'addiction':ab,ti) OR 'drug dependence':ab,ti OR 'alcohol addiction':ab,ti OR 'opioid addiction':ab,ti OR 'cocaine addiction':ab,ti OR 'methamphetamine addiction':ab,ti OR 'cannabis addiction':ab,ti) AND ('machine learning':ab,ti OR 'deep learning':ab,ti OR 'neural network':ab,ti OR 'supervised learning':ab,ti OR 'predictive modeling':ab,ti OR 'supervised learning':ab,ti OR 'support vector machine':ab,ti OR 'random forest':ab,ti OR 'gradient boosting':ab,ti) AND ('Readmission':ab,ti OR 'Rehospitalization':ab,ti OR 'Relapse':ab,ti OR 'treatment outcome':ab,ti OR 'Severity':ab,ti)

Table S1: Detailed list of excluded studies

**Authors**	**Year**	**Title**	**Reason**
Montoya, I.D.	2024	New strategies for medications to treat substance use disorders	Review
Banerjee, S.	2024	Trajectories of remitted psychotic depression: identification of predictors of worsening by machine learning	No SUD
Broomer, M.C.	2024	Examining a punishment-related brain circuit with miniature fluorescence microscopes and deep learning	Animal model
Yadav, T.	2024	Pilot study with randomised control of dual site theta burst transcranial magnetic stimulation (TMS) for methamphetamine use disorder: a protocol for the TARTAN study	Study Protocol
Bejerholm, U.	2024	Individual Placement and Support for persons with alcohol and drug addiction in a Swedish context (IPS-ADAS): study protocol for a randomised controlled trial	Study Protocol
Amanollahi, M.	2024	Machine learning applied to the prediction of relapse, hospitalization, and suicide in bipolar disorder using neuroimaging and clinical data: A systematic review	Review
Harp, N.R.	2024	Neuromarkers in addiction: definitions, development strategies, and recent advances	Review
Carreiro, S.	2024	Evaluation of a digital tool for detecting stress and craving in SUD recovery: An observational trial of accuracy and engagement	No ML
Winter, N.R.	2024	A Systematic Evaluation of Machine Learning-Based Biomarkers for Major Depressive Disorder	No SUD
Javorski, M.J.	2024	Infective Endocarditis in Patients Addicted to Injected Opioid Drugs	Wrong Outcome
Mestre-Bach, G.	2023	Neural mechanisms linked to treatment outcomes and recovery in substance-related and addictive disorders	Review
Pettorruso, M.	2023	Predicting outcome with Intranasal Esketamine treatment: A machine-learning, three-month study in Treatment-Resistant Depression (ESK-LEARNING)	No SUD
Infanti, A.	2023	Gaming passion contributes to the definition and identification of problematic gaming	No SUD
Desai, K.	2023	Development of Machine Learning-Based Models to Predict Short Term Success in Substance Use Disorder treatment	Conference abstract
Baurley, J.W.	2023	Prediction of drinking behaviors around treatment for alcohol use disorder	Conference abstract
Xu, J.	2023	Using machine learning to identify factors related to nitrous oxide (laughing gas) relapse among adolescents	Wrong publication type
Xu, J.	2023	Using machine learning to identify factors related to nitrous oxide (laughing gas) relapse among adolescents	No SUD sample
Peng, Q.	2023	Pleiotropic loci for cannabis use disorder severity in multi-ancestry high-risk populations	Vitro
Yang, L.	2023	Machine learning with neuroimaging biomarkers: Application in the diagnosis and prediction of drug addiction	Review
Wilkinson, C.S.	2023	Listening to the Data: Computational Approaches to Addiction and Learning	Non empirical paper

Authors	Year	Title	Reason
Infanti, A.	2023	Gaming passion contributes to the definition and identification of problematic gaming	No SUD
Infanti, A.	2023	How gaming passion contributes to the definition and diagnosis of problem gaming: A combined person-centered and supervised machine-learning analysis	No SUD
Morawetz, C.	2023	Mood Variability, Craving, and Substance Use Disorders: From Intrinsic Brain Network Connectivity to Daily Life Experience	No ML
Curtis, B.	2023	AI-based analysis of social media language predicts addiction treatment dropout at 90 days	Social media based
Wang, Z.	2023	Editorial: Neural pathophysiological mechanisms of neuropsychiatric disorders underlying substance abuse	Non empirical paper
Soares-Cunha, C.	2023	Ventral pallidal regulation of motivated behaviors and reinforcement	Review
Antons, S.	2023	Connectome-based prediction of craving in gambling disorder and cocaine use disorder	No SUD
Ruan, X.	2023	Alterations of brain activity in patients with alcohol use disorder: a resting-state fMRI study	No ML
Sajjadian, M.	2023	Prediction of depression treatment outcome from multimodal data: a CAN-BIND-1 report	Review
Phaterpekar, T.	2023	Machine Learning Prediction of Quality of Life Improvement During Antidepressant Treatment of Patients With Major Depressive Disorder: A STAR*D and CAN-BIND-1 Report	No SUD
Mehrpour, O.	2023	Outcome prediction of methadone poisoning in the United States: implications of machine learning in the National Poison Data System (NPDS)	NO DSM/ICD
Curtis, B.	2023	AI-based analysis of social media language predicts addiction treatment dropout at 90 days	Social media based
Del Fabro, L.	2023	Machine learning methods to predict outcomes of pharmacological treatment in psychosis.	No SUD sample
Maes, M.	2023	Research and Diagnostic Algorithmic Rules (RADAR) for mood disorders, recurrence of illness, suicidal behaviours, and the patient's lifetime trajectory.	No SUD sample
Nordin, N.	2023	An explainable predictive model for suicide attempt risk using an ensemble learning and Shapley Additive Explanations (SHAP) approach	No SUD sample
Takano, A.	2023	Wearable Sensor and Mobile App-Based mHealth Approach for Investigating Substance Use and Related Factors in Daily Life	Study Protocol
Volkow, N.D.	2023	Substance use disorders: a comprehensive update of classification, epidemiology, neurobiology, clinical aspects, treatment and prevention	Non empirical paper
Ding, X.	2023	The effect of repetitive transcranial magnetic stimulation on electroencephalography microstates of patients with heroin-addiction	No ML
Dokkedal-Silva, V.	2023	Neural and functional connectivity changes caused by long-term use of benzodiazepines: Investigating the mechanisms of tolerance and dependence	Non empirical paper
Hassan, M.M.	2023	An efficient Apriori algorithm for frequent pattern in human intoxication data	NO DSM/ICD
Slosky, L.M.	2022	Establishment of multi-stage intravenous self-administration paradigms in mice	Animal Model
Goldstein, R.	2022	Natural Language Processing of Movie Recall and Drug Fluency, and Associated Brain Function, in Cocaine and Heroin Addiction	No ML
Jiang, C.	2022	Chloral Hydrate Alters Brain Activation Induced by Methamphetamine-Associated Cue and Prevents Relapse	No ML
Liu, T.	2022	Linguistic predictors from Facebook postings of substance use disorder treatment retention versus discontinuation.	Social media based

Authors	Year	Title	Reason
Lin, X.	2022	Connectome-based predictive modelling of smoking severity in smokers.	No ML
Lissemore, J. I.	2022	Transcranial Magnetic Stimulation Indices of Cortical Excitability Enhance the Prediction of Response to Pharmacotherapy in Late-Life Depression.	No SUD sample
Kusudo, K	2022	Decision tree classification of cognitive functions with D(2) receptor occupancy and illness severity in late-life schizophrenia.	Non empirical paper
Heinz, A.	2022	AIM in Alcohol and Drug Dependence	Non empirical paper
Bouhadja, A.	2022	A Review on Recent Machine Learning Applications for Addiction Disorders	Review
Camchong, J.	2022	Resting Hypoconnectivity of Theoretically Defined Addiction Networks during Early Abstinence Predicts Subsequent Relapse in Alcohol Use Disorder	No ML
Tolomeo, S.	2022	Brain network dysfunctions in addiction: a meta-analysis of resting-state functional connectivity	Review
Machetanz, L.	2022	Model Building in Forensic Psychiatry: A Machine Learning Approach to Screening Offender Patients with SSD	No SUD sample
Huber, M.	2022	Machine Learning for Outcome Prediction in First-Line Surgery of Prolactinomas	No SUD sample
Yuan, L.	2022	Abnormal Brain Network Interaction Associated With Positive Symptoms in Drug-Naive Patients With First-Episode Schizophrenia	No SUD sample
Lichenstein, S.	2022	Identification and External Validation of a Problem Cannabis Use Brain Network	No SUD sample
Schwebel, F.J.	2022	Using Decision Trees to Identify Salient Predictors of Cannabis-Related Outcomes	No SUD
Selamat, N.A.	2022	Association features of smote and rose for drug addiction relapse risk	No SUD
Slosky, L.M.	2022	Establishment of multi-stage intravenous self-administration paradigms in mice	Animal model
Bossarte, R.M.	2022	The Appalachia Mind Health Initiative (AMHI): a pragmatic randomized clinical trial of adjunctive internet-based cognitive behavior therapy for treating major depressive disorder among primary care patients	No SUD sample
Saadatmand, S.	2022	Predicting the necessity of oxygen therapy in the early stage of COVID-19 using machine learning	No SUD sample
Janiri, D.	2022	Editorial: Late-onset depression and mania: Diagnosis, treatment and life events as risk factors	Non empirical paper
Nimitvilai-Roberts, S.	2022	The ethanol inhibition of basolateral amygdala neuron spiking is mediated by a λ-aminobutyric acid type A-mediated tonic current	Vitro
Eddie, D.	2022	Closing the brain-heart loop: Towards more holistic models of addiction and addiction recovery	No ML
McIntyre, R.S.	2022	The clinical characterization of the adult patient with bipolar disorder aimed at personalization of management	No SUD sample
Malhotra, D.K.	2022	The Role of Artificial Intelligence (AI) in Assisting Applied Natya Therapy for Relapse Prevention in De-addiction	Non empirical paper
Kunas, S.L.	2022	Neurofunctional Alterations of Cognitive Down-Regulation of Craving in Quitting Motivated Smokers	No ML
Curtis, B.L.	2021	Digital markers for forecasting relapse & recovery	Conference presentation
Smith, A.	2021	A “master regulator” of opioid reward and aversion in the ventromedial prefrontal cortex	Animal Model
Hinds, N.	2021	Neural activity within distinct subregions of the periaqueductal gray is associated with social Vs. Nonsocial Stress-induced cocaine seeking in rats	Animal Model

Authors	Year	Title	Reason
Ballester, P.L.	2021	Accelerated brain aging in major depressive disorder and antidepressant treatment response: A CAN-BIND report.	No SUD sample
Kosciolek, T.	2021	Individuals with substance use disorders have a distinct oral microbiome pattern	No SUD
Peng, Q.	2021	Common genetic substrates of alcohol and substance use disorder severity revealed by pleiotropy detection against GWAS catalog in two populations	No SUD
Moshontz, H.	2021	Prospective Prediction of Lapses in Opioid Use Disorder: Protocol for a Personal Sensing Study	Study Protocol
Weigard, A.S.	2021	Evidence accumulation and associated error-related brain activity as computationally-informed prospective predictors of substance use in emerging adulthood.	No ML
Cochran, G.	2021	Validation and threshold identification of a prescription drug monitoring program clinical opioid risk metric with the WHO alcohol, smoking, and substance involvement screening test.	No SUD sample
Chen, Po-Ku	2021	Anti-TROVE2 Antibody Determined by Immune-Related Array May Serve as a Predictive Marker for Adalimumab Immunogenicity and Effectiveness in RA.	Vitro
Li, Xiao-Yan	2021	Identifying clinical risk factors correlate with suicide attempts in patients with first episode major depressive disorder.	No SUD sample
Smith, R.	2021	Greater decision uncertainty characterizes a transdiagnostic patient sample during approach-avoidance conflict: A computational modelling approach	No ML
Le Glaz, A.	2021	Machine learning and natural language processing in mental health: Systematic review	Review
Steele, V.R.	2021	Treating cocaine and opioid use disorder with transcranial magnetic stimulation: A path forward	No ML
Gelbard, R.B.	2021	An integrative model using flow cytometry identifies nosocomial infection after trauma	Vitro
Zhao, Y.	2021	Anterior Cingulate Cortex in Addiction: New Insights for Neuromodulation	Review
Ker, S.	2021	Factors that affect patient attrition in buprenorphine treatment for opioid use disorder: A retrospective real-world study using electronic health records	No ML
Kuntz, A.	2021	Persistence of Neuronal Alterations in Alcohol-Dependent Patients at Conclusion of the Gold Standard Withdrawal Treatment: Evidence From ERPs	No ML
Shah, D.	2021	Using a machine learning approach to investigate factors associated with treatment-resistant depression among adults with chronic non-cancer pain conditions and major depressive disorder	No SUD sample
Jha, D.	2021	Identifying and Characterizing Opioid Addiction States Using Social Media Posts	Social media based
Irving, J.	2021	Using Natural Language Processing on Electronic Health Records to Enhance Detection and Prediction of Psychosis Risk	SUD not main variable
Beacher, N.J.	2021	Circuit Investigation of Social Interaction and Substance Use Disorder Using Miniscopes	No ML
Smith, R.	2021	Perceptual insensitivity to the modulation of interoceptive signals in depression, anxiety, and substance use disorders	No treatment outcome
Guttha, N.	2021	Towards the Development of a Substance Abuse Index (SEI) through Informatics	No SUD sample
Davis-Martin, RE.	2021	Alcohol Use Disorder in the Age of Technology: A Review of Wearable Biosensors in Alcohol Use Disorder Treatment	Review
Oliver, J.	2020	Exposure to smoking context potentiates habitual motor response	Conference presentation

Authors	Year	Title	Reason
Hu, Z.	2020	Analysis of substance use and its outcomes by machine learning: II. Derivation and prediction of the trajectory of substance use severity	No SUD
Hsu, M.	2020	Digital Phenotyping to Enhance Substance Use Treatment During the COVID-19 Pandemic	Review
Tomasi, D.	2020	Accelerated aging of the amygdala in alcohol use disorder: Implications for the 'dark-side' of addiction	Conference presentation
Keyes, P.C.	2020	Orchestrating Opiate-Associated Memories in Thalamic Circuits	No ML
Xu, B.	2020	Machine Learning Analysis of Electronic Nose in a Transdiagnostic Community Sample With a Streamlined Data Collection Approach: No Links Between Volatile Organic Compounds and Psychiatric Symptoms	NO DSM/ICD
Roglio, V.S.	2020	Prediction of attempted suicide in men and women with crack-cocaine use disorder in Brazil	No ML
Barenholtz, E.	2020	Machine-learning approaches to substance-abuse research: emerging trends and their implications.	Review
Habelt, B.	2020	Biomarkers and neuromodulation techniques in substance use disorders.	Review
Lauvsnes, A.D.F.	2020	Mobile Sensing in Substance Use Research: A Scoping Review.	Review
Yip, S.W.;	2020	Toward Addiction Prediction: An Overview of Cross-Validated Predictive Modeling Findings and Considerations for Future Neuroimaging Research.	Review
Zhdanov, A.	2020	Use of Machine Learning for Predicting Escitalopram Treatment Outcome From Electroencephalography Recordings in Adult Patients With Depression.	No SUD sample
Larivière, S.	2020	Functional connectome contractions in temporal lobe epilepsy: Microstructural underpinnings and predictors of surgical outcome.	No SUD sample
Arenas-Castañeda, P.E.	2020	Universal mental health screening with a focus on suicidal behaviour using smartphones in a Mexican rural community: protocol for the SMART-SCREEN population-based survey.	NO DSM/ICD
Rashid, B.	2020	Towards a brain-based predictome of mental illness	Review
Hayes, A.	2020	The neurobiology of substance use and addiction: Evidence from neuroimaging and relevance to treatment	No ML
Hagerty, S.L.	2020	DRD2 methylation is associated with executive control network connectivity and severity of alcohol problems among a sample of polysubstance users	No ML
Senior, M.	2020	Identifying Predictors of Suicide in Severe Mental Illness: A Feasibility Study of a Clinical Prediction Rule (Oxford Mental Illness and Suicide Tool or OxMIS)	SUD not main variable
Droutman, V.	2019	Neurocognitive decision-making processes of casual methamphetamine users	No ML
Foster, S.	2019	Estimating patient-specific treatment advantages in the 'Treatment for Adolescents with Depression Study'.	No SUD sample
Mithani, K.	2019	Connectomic Profiling Identifies Responders to Vagus Nerve Stimulation.	No SUD sample
Ekhtiari, H.	2019	Physical characteristics not psychological state or trait characteristics predict motion during resting state fMRI.	SUD not main variable
Jonas, B.	2019	Predictors of treatment response in a web-based intervention for cannabis users	NO DSM/ICD
Brady, K.T.	2019	Improving our understanding of substance use disorders	Non empirical paper
Edgcomb, J.	2019	High-Risk Phenotypes of Early Psychiatric Readmission in Bipolar Disorder With Comorbid Medical Illness	No SUD sample

Authors	Year	Title	Reason
Symons, M.	2019	Machine learning vs addiction therapists: A pilot study predicting alcohol dependence treatment outcome from patient data in behavior therapy with adjunctive medication	No SUD
Steele, J.D.	2019	Pragmatic neuroscience for clinical psychiatry	Review
Zilcha-Mano, S.	2019	Are there any robust predictors of ”sudden gainers,” and how is sustained improvement in treatment outcome achieved following a gain?	No SUD sample
-	2019	7th International Conference for Smart Health, ICSH 2019	Conference presentation
Morris, L.S.	2018	Naltrexone ameliorates functional network abnormalities in alcohol-dependent individuals	No ML
Suvisaari, J.	2018	Is It Possible to Predict the Future in First-Episode Psychosis?	SUD not main variable
Maciukiewicz, M.	2018	GWAS-based machine learning approach to predict duloxetine response in major depressive disorder.	No SUD sample
Kornfield, R.	2018	Detecting Recovery Problems Just in Time: Application of Automated Linguistic Analysis and Supervised Machine Learning to an Online Substance Abuse Forum.	Social media based
Liu, J.-F.	2018	Drug addiction: a curable mental disorder?	No ML
Kedzior, K.K.	2018	Can deep transcranial magnetic stimulation (DTMS) be used to treat substance use disorders (SUD)? A systematic review	Review
Reece, A.S.	2018	Pathways from epigenomics and glycobiology towards novel biomarkers of addiction and its radical cure	Review
Kautzky, A.	2018	Refining Prediction in Treatment-Resistant Depression: Results of Machine Learning Analyses in the TRD III Sample	No SUD sample
Clausen, A.	2017	Assessing relationships between childhood trauma and brain morphology in large community-based adult sample	SUD not main variable
Zilverstand, A.	2017	Resting-state connectivity defines neurobiological subtypes underlying different personality profiles in cocaine addiction	Conference presentation
Kober, H.	2017	Meta-analysis of drug cue reactivity: Towards neuromarkers of craving and relapse	Review
Murphy, A.	2017	Acute D3 Antagonist GSK598809 Selectively Enhances Neural Response during Monetary Reward Anticipation in Drug and Alcohol Dependence	No ML
Chekroud, A.M.	2017	Reevaluating the Efficacy and Predictability of Antidepressant Treatments: A Symptom Clustering Approach.	No SUD sample
Thomas, K.	2017	Psilocybin-Assisted Therapy: A Review of a Novel Treatment for Psychiatric Disorders.	Review
Forster, S.E.	2017	Neural responses to negative outcomes predict success in community-based substance use treatment.	No ML
Kohno, M.	2017	Executive control and striatal resting-state network interact with risk factors to influence treatment outcomes in alcohol-use disorder	No ML
Wei, Z.	2017	Summary and prospect	Non empirical paper
Avram, M.J.	2017	Journal-related Activities and Other Special Activities at the 2017 American Society of Anesthesiologists Meeting	Non empirical paper
Kautzky, A	2017	A New Prediction Model for Evaluating Treatment-Resistant Depression	No SUD sample
Li, C.-S.	2016	Cocaine dependence and thalamic functional connectivity: A multivariate pattern analysis	Conference presentation
Liu, T.	2016	Altered Long- and Short-Range Functional Connectivity in Patients with Betel Quid Dependence: A Resting-State Functional MRI Study	No ML
Lim, T.V.	2016	Endogenous opioid blockade modulates neural networks implicated in attentional and behavioural control in individuals recovering from alcohol and drug dependence	No ML

Authors	Year	Title	Reason
Venkiteswaran, K.	2016	Transplantation of human retinal pigment epithelial cells in the nucleus accumbens of cocaine self-administering rats provides protection from seeking	Vitro
Wei, Z.	2016	Resting-state functional connectivity between the dorsal anterior cingulate cortex and thalamus is associated with risky decision-making in nicotine addicts.	No ML
Lam, Raymond W.	2016	Discovering biomarkers for antidepressant response: protocol from the Canadian biomarker integration network in depression (CAN-BIND) and clinical characteristics of the first patient cohort.	No SUD sample
Blasco, M.J.	2016	Predictive models for suicidal thoughts and behaviors among Spanish University students: Rationale and methods of the UNIVERSAL (University & mental health) project	No SUD sample
Huys, Q.J.M.	2016	Computational Psychiatry: From Mechanistic Insights to the Development of New Treatments	Non empirical paper
Voon, V.	2015	Jumping the gun: Mapping a translational network in waiting impulsivity	Conference presentation
Spanagel, R.	2015	A systems medicine approach towards understanding alcohol addiction	Conference presentation
Savulich, G.	2015	Themodulatory effects of naltrexone on the underlying neural network in alcohol and drug dependence: An fMRI study	No ML
Blum, K.	2015	Reward Deficiency Syndrome Solution System™ (RDSS): A 50-year sojourn	No ML
Schäfer, J.	2015	Predicting smoking cessation and its relapse in HIV-infected patients: The Swiss HIV Cohort Study	No ML
Nevidimova, T.I.	2015	Association between pathological olfaction, immunological abnormalities and predisposition to substance use	NO DSM/ICD
Gowin, J.L.	2015	Individualized relapse prediction: Personality measures and striatal and insular activity during reward-processing robustly predict relapse.	No ML
Luo, Sean X.	2015	Toward personalized smoking-cessation treatment: Using a predictive modeling approach to guide decisions regarding stimulant medication treatment of attention-deficit/hyperactivity disorder (ADHD) in smokers.	SUD not main variable
Tanana, M.	2015	Recursive Neural Networks for Coding Therapist and Patient Behavior in Motivational Interviewing	No SUD
Morein-Zamir, S.	2015	Fronto-striatal circuits in response-inhibition: Relevance to addiction	No ML
Moorman, D.E.	2015	Differential roles of medial prefrontal subregions in the regulation of drug seeking	No ML
Stein, E.	2014	Imaging biomarkers of addiction: From predicting use status to treatment outcome	Review
Kilts, C.D.	2014	Individual differences in attentional bias associated with cocaine dependence are related to varying engagement of neural processing networks	No ML
Whelan, R.	2014	When optimism hurts: inflated predictions in psychiatric neuroimaging.	No SUD sample
Luo, G.	2014	A roadmap for designing a personalized search tool for individual healthcare providers	No SUD sample
Kiang, M.	2013	Association of abnormal semantic processing with delusion-like ideation in frequent cannabis users: An electrophysiological study	No ML
Schmidt, H.D.	2013	Epigenetics and psychostimulant addiction	No ML
Cysique, L.A.	2011	Prevalence of non-confounded HIV-associated neurocognitive impairment in the context of plasma HIV RNA suppression	No SUD sample
Qi, Y.B.	2011	Altered functional connectivity of prefrontal cortex in chronic heroin abusers	No ML

Authors	Year	Title	Reason
Hester, R.	2010	The role of executive control in human drug addiction	Non empirical paper
Boyer, E.W.	2010	Wireless Technologies, Ubiquitous Computing and Mobile Health: Application to Drug Abuse Treatment and Compliance with HIV Therapies	Non empirical paper
Verdejo-García, A.	2006	Emotion, decision-making and substance dependence: A somatic-marker model of addiction	No ML
Dong, Z.F.	2006	Morphine conditioned place preference depends on glucocorticoid receptors in both hippocampus and nucleus accumbens	Vitro
Azorin, J.-M.	2004	Behavioral sensitization models and mental disorders: Current issues	Review
Giuffredi, C.	2003	Alcohol addiction: Evaluation of alcohol abstinence after a year of psycho-medical-social treatment	No ML
Buscema, M.	2002	A brief overview and introduction to artificial neural networks	Review
Pich, E.M.	1998	Neural substrate of nicotine addiction as defined by functional brain maps of gene expression	No ML
Baker, T.B.	1986	The motivation to use drugs: a psychobiological analysis of urges.	No ML

Table S2: Key items of the CHARMS checklist and reporting per item

Key Items	Davis 2021	Davis 2022	Bailey 2022	Yip 2019	Nasir 2021	Steele 2018	Gottlieb 2022	Acion 2017	Koban 2023	Symons 2020	Morel 2020	Shrestha 2023	Cox 2020	Jing 2020	Cavicchioli 2021	Suchting 2019	Costello 2021	Steele 2014	Annis 2022	Kang 2022	Roberts 2022	Baucum 2023	Burgess-Hull 2023	Houghton 2023	Eddie 2024	Heberle 2024
Study dates	Y	Y	Y	N	Y	N	Y	Y	Y	N	Y	N	Y	N	Y	Y	Y	N	Y	N	Y	N	Y	N	Y	Y
Was the same outcome definition (and method for measurement) used in all patients?	Y	Y	Y	Y	Y	Y	Y	N	Y	Y	Y	N	Y	Y	Y	Y	Y	Y	Y	Y	Y	Y	Y	N	Y	Y
Type of outcome (e.g., single or combined endpoints/outcomes).	single	single	single	single	combined	single	single	single	combined	combined	single	single	single	single	combined	single	single	single	single	combined	single	single	single	combined	single	single
Was the outcome assessed without knowledge of the candidate predictors?	Y	Y	Y	Y	Y	Y	Y	Y	Y	Y	Y	Y	Y	Y	Y	Y	Y	Y	Y	Y	Y	Y	Y	Y	Y	Y
Were candidate predictors part of the outcome?	N	N	N	N	N	N	N	N	N	N	N	N	N	N	N	N	N	N	N	N	N	N	N	N	N	N
Time of outcome occurrence or summary of duration of follow-up?	Y	Y	N	Y	N	Y	Y	Y	Y	Y	Y	Y	N	Y	Y	Y	Y	Y	N	N	Y	Y	N	N	Y	Y
Timing of predictor measurement	Y	Y	N	Y	Y	Y	Y	Y	Y	Y	Y	Y	N	Y	Y	Y	Y	Y	Y	Y	Y	Y	Y	N	Y	Y
Were predictors assessed blinded for outcome, and for each other?	Y	Y	Y	Y	Y	Y	Y	Y	N	Y	Y	Y	Y	Y	Y	N	Y	Y	Y	Y	Y	Y	Y	Y	Y	Y
Handling of predictors in the modelling	both	both	both	both	both	both	both	both	continuous	both	both	continuous	both	both	both	both	both	both	both	both	both	both	both	both	both	both
Number of participants with any missing value	N	N	N	Y	N	Y	N	Y	Y	N	Y	N	N	N	N	N	Y	N	N	N	N	Y	Y	N	N	N
Number of participants with missing data for each predictor handling of missing data	Y	Y	N	Y	N	N	N	Y	Y	N	Y	N	N	N	N	N	N	N	N	N	Y	Y	Y	Y	N	Y
Method for selection of predictors for inclusion in multivariable modelling	Y	Y	Y	N	N	Y	Y	Y	Y	N	N	Y	Y	N	Y	Y	N	N	Y	Y	Y	N	Y	Y	N	Y
Method for selection of predictors during multivariable modelling and criteria used	Y	N	Y	N	Y	Y	Y	Y	Y	Y	N	N	Y	Y	N	Y	N	Y	Y	Y	Y	N	Y	N	N	N
Shrinkage of predictor weights or regression coefficients (e.g., N shrinkage,uniform shrinkage, penalized estimation)	N	N	N	N	N	N	N	N	N	N	N	N	N	N	Y	N	N	N	N	N	N	N	Y	N	N	N
Calibration and Discrimination measures with confidence intervals	N	N	Y	Y	Y	N	N	Y	Y	Y	Y	Y	N	Y	Y	Y	N	Y	Y	Y	Y	Y	Y	Y	N	Y
Classification measures and whether a priori cut points were used	N	Y	Y	Y	Y	Y	Y	N	Y	Y	N	Y	N	N	Y	N	N	Y	Y	Y	Y	N	Y	Y	Y	Y
In case of poor validation, whether model was adjusted or updated	N	N	N	N	N	N	N	N	N	N	N	N	N	N	N	N	N	N	N	N	N	N	N	N	N	N
Results Final and other multivariable models	Y	Y	N	N	Y	N	Y	Y	N	Y	Y	N	N	Y	Y	Y	N	Y	Y	Y	Y	Y	Y	Y	Y	Y
Any alternative presentation of the final prediction models, e.g., sum score, Nmogram	N	Y	N	Y	N	N	N	N	Y	Y	Y	N	N	N	N	Y	N	N	Y	Y	Y	Y	N	N	N	N
Comparison of the distribution of predictors (including missing data) for development and validation datasets	N	N	N	N	N	N	N	N	N	Y	N	N	N	N	N	N	N	N	N	N	N	N	N	N	N	N
Interpretation of presented models	Y	Y	N	N	Y	Y	Y	N	Y	Y	Y	N	N	N	Y	Y	N	Y	Y	N	Y	Y	Y	Y	Y	Y
Comparison with other studies, discussion of generalizability, strengths and limitations	N	N	N	N	N	N	N	N	N	Y	Y	N	N	N	N	N	N	N	N	N	N	N	N	N	N	N

Notes:We've considered the reporting of AUC or AUROC to be adequate reporting for the Calibration key item. CHARMS framework is unsuitable for assessing clustering algorithms, which are used in unsupervised learning without external labels for validation. Therefore, this instrument was not used to assess the quality of the work of Panlilio et al. 2020 and Burgess-Hull et al. 2022.

Table S3: Key items of the MI-CLAIM checklist and reporting per item

Key Items	Davis 2021	Davis 2022	Bailey 2022	Yip 2019	Nasir 2021	Steele 2018	Gottlieb 2022	Acion 2017	Koban 2023	Symons 2020	Morel 2020	Shrestha 2023	Cox 2020	Jing 2020	Cavicchioli 2021	Suchting 2019	Costello 2021	Steele 2014	Annis 2022	Kang 2022	Roberts 2022	Baucum 2023	Burgess-Hull 2023	Houghton 2023	Eddie 2024	Heberle 2024
The clinical problem in which the model will be employed is clearly detailed in the paper.	Y	Y	Y	Y	Y	Y	Y	Y	Y	Y	Y	Y	Y	Y	Y	Y	Y	Y	Y	Y	Y	Y	Y	Y	Y	Y
The research question is clearly stated.	Y	Y	Y	Y	Y	Y	Y	Y	Y	Y	Y	Y	N	Y	Y	Y	Y	Y	Y	Y	Y	Y	Y	Y	Y	Y
The characteristics of the cohorts (training and test sets) are detailed in the text.	N	N	N	N	N	N	N	Y	N	Y	Y	N	N	N	N	N	N	N	N	N	N	Y	N	N	N	Y
The cohorts (training and test sets) are shown to be representative of real-world clinical settings.	N	N	N	N	Y	N	N	Y	Y	Y	Y	N	N	N	N	N	Y	N	Y	N	Y	Y	N	N	Y	N
The state-of-the-art solution used as a baseline for comparison has been identified and detailed.	N	N	N	N	N	N	N	N	N	N	Y	N	N	N	N	N	N	N	N	N	N	N	N	N	N	N
The origin of the data is described and the original format is detailed in the paper.	Y	Y	Y	Y	Y	Y	Y	Y	Y	Y	Y	Y	N	N	Y	Y	Y	Y	Y	Y	Y	Y	Y	Y	Y	Y
Transformations of the data before it is applied to the proposed model are described	Y	Y	N	Y	N	Y	Y	N	Y	N	N	Y	Y	Y	Y	Y	Y	Y	N	Y	Y	Y	Y	Y	Y	Y
The independence between training and test sets has been proven in the paper.	N	N	N	Y	N	Y	Y	N	Y	Y	Y	N	N	N	Y	N	N	Y	N	N	Y	N	Y	Y	Y	Y
Details on the models that were evaluated and the code developed to select the best model are provided.	N	N	Y	N	N	N	N	N	Y	N	N	N	Y	N	N	N	N	N	N	N	Y	N	Y	N	N	Y
Is the input data type structured or unstructured? (prov always structured)	Structured	Structured	Structured	Structured	Structured	Structured	Structured	Structured	Structured	Structured	Structured	Structured	Structured	Structured	Structured	Structured	Structured	Structured	Structured	Structured	Structured	Structured	Structured	Structured	Structured	Structured
The primary metric selected to evaluate algorithm performance (e.g., AUC, F-score, etc.), including the justification for selection, has been clearly stated	N	N	N	N	Y	N	N	Y	Y	Y	Y	Y	N	Y	N	Y	N	Y	Y	Y	Y	N	N	Y	N	Y
The primary metric selected to evaluate the clinical utility of the model (e.g., ppV, NNT, etc.), including the justification for selection, has been clearly stated.	N	N	N	N	N	Y	N	N	N	N	N	Y	N	N	N	N	N	Y	N	N	Y	N	N	N	N	N
The performance comparison between baseline and proposed model is presented with the appropriate statistical significance.	N	N	N	N	N	N	N	Y	N	Y	Y	N	N	N	N	N	N	N	N	N	N	Y	N	N	N	N
Examination technique 1	Y	Y	N	Y	Y	Y	Y	N	Y	Y	Y	N	N	Y	Y	Y	N	N	N	Y	Y	N	Y	Y	Y	Y
Examination technique 2	N	N	N	N	N	N	N	N	Y	N	N	N	N	Y	N	N	N	N	N	N	N	N	N	N	N	Y
A discussion of the relevance of the examination results with respect to model/algorithm performance is presented.	N	Y	Y	N	Y	N	Y	Y	Y	Y	Y	N	N	N	Y	Y	N	Y	Y	N	Y	Y	Y	N	N	N
A discussion of the feasibility and significance of model interpretability at the case level if examination methods are uninterpretable is presented.	N	N	N	N	N	N	N	N	N	N	N	N	N	N	N	N	N	N	N	N	N	N	Y	N	N	N
A discussion of the reliability and robustness of the model as the underlying data distribution shifts is included.	N	N	N	N	N	N	N	N	N	N	N	N	N	N	N	N	N	N	N	N	Y	N	N	N	N	N
Reproducibility: choose appropriate tier of transparency	Tier 4	Tier 4	Tier 1	Tier 4	Tier 4	Tier 4	Tier 4	Tier 4	Tier 1	Tier 4	Tier 4	Tier 4	Tier 1	Tier 4	Tier 4	Tier 4	Tier 4	Tier 4	Tier 4	Tier 4	Tier 1	Tier 4	Tier 4	Tier 4	Tier 4	Tier 1

Notes: Tier 1: complete sharing of the code; Tier 2: allow a third party to evaluate the code for accuracy/fairness; share the results of this evaluation; Tier 3: release of a virtual machine (binary) for running the code on new data without sharing its details; Tier 4: no sharing. MI-CLAIM framework is unsuitable for assessing clustering algorithms, which are used in unsupervised learning without external labels for validation. Therefore, this instrument was not used to assess the quality of the work of Panlilio et al. 2020 and Burgess-Hull et al. 2022.
